# A feasibility study of the costs and consequences of improving the oral health of older people in care homes: findings from the TOPIC study

**DOI:** 10.1186/s12903-025-06322-6

**Published:** 2025-07-02

**Authors:** Ciaran O’Neill, Paul R. Brocklehurst, Saif Syed, Michelle Harvey, Sana Daniyal, Sinead Watson, Nia Goulden, Anna Verey, Peter Cairns, Anja Heilmann, Zoe Hoare, Frank Kee, Joe Langley, Nat Lievesley, Andrea Sherriff, Craig J. Smith, Rebecca R. Wassall, Richard G. Watt, Gerald McKenna, Georgios Tsakos

**Affiliations:** 1 Centre for Public Health, Queens University Belfast, Belfast, UK; 2 Public Health Wales, Cardiff, UK; 3 Institute of Epidemiology and Health Care, University College London, London, UK; 4 Institute of Epidemiology & Health Care, University College London, London, UK; 5 Centre for Public Health, Queens University Belfast, Belfast, UK; 6 North Wales Organisation for Randomised Trials in Health (NWORTH), Bangor University, Bangor, UK; 7 Institute of Psychiatry, Psychology, and Neuroscience, King’s College London, London, UK; 8 Public contributor (PPI), Northern Ireland, UK; 9 North Wales Medical School, Bangor University, Bangor, UK; 10 Sheffield Hallam University, Sheffield, UK; 11 Public contributor (PPI), London, UK; 12 School of Medicine, University of Glasgow, Glasgow, UK; 13 Division of Cardiovascular Sciences, University of Manchester, Manchester, UK; 14 School of Dental Sciences, Newcastle University, Newcastle, UK

**Keywords:** Oral health, Older people, Care homes, Feasibility study, Economics evaluation

## Abstract

**Background:**

In the UK older adults in care homes have exhibited poorer oral health than age-equivalent persons in the community. In response the National Institute for Health and Care Excellence issued guidance (NG48) on the maintenance and improvement of oral health in this group. Currently, there is little evidence on the cost-effectiveness of such interventions. The aim of this study was to examine the feasibility of evaluating an intervention framed around NICE guidance.

**Methods:**

The study was undertaken in 22 care homes across 2 sites with eligibility criteria used to ensure access to residents for whom the oral health care intervention was relevant and who could consent to participation. The intervention followed the guidance issued by NICE in respect of care staff knowledge; oral health assessment and development of care plans, and provision of daily mouth care to residents. Quantitative and qualitative data were collected from residents and care home managers and interviews undertaken with a range of stakeholders. Quantitative data from residents comprised EQ5D5L at baseline, 6 and 12 months, qualitiative data was taken from interviews. Descriptive statistics and a discussion of themes raised in surveys and interviews was undertaken. The trial was registered with the UK’s Clinical Study Registry (ISRCTN10276613) on 17/04/2020.

**Results:**

Of 119 residents recruited, 115 provided usable EQ5D5L data at baseline. The data had good face validity. Managers from 7 of 22 homes provided responses to the care home survey. All responding care homes routinely recorded information on care provided to residents and 5 of the 7 recorded information on the funding source for that care. Care assistant time was a key consideration among managers in terms of resource use. Residents overall quality of life was a key consideration among managers in terms of outcomes. Among key stakeholders, there was a universal appreciation of the need to improve the evidence base on the value for money of interventions framed around NG48.

**Conclusion:**

The study supports the case for the conduct of an economic evaluation in a definitive trial to address a manifest gap in the evidence base on oral hygiene interventions in this context.

Good oral health is important to eating and social interaction and is associated with better health-related quality of life ratings [1, 2]. Among older persons (those aged over 65) specifically, good oral health has been shown to improve oral health-related quality of life [3]general health [4] and diet [5, 6]. While significant improvements in the oral health of older persons have been recorded in the UK over time [7]variations are evident across groups of older persons with respect to age and dependency level. Those who are older and in particular people who live in care homes, for example, have been shown to exhibit poorer oral health compared to those living in the community [8]. This can in part be explained by their more complex needs and higher levels of care dependency [9] making self-care related to oral hygiene more difficult. Diets rich in sugars [10]polypharmacy resulting in dry mouth [11] and barriers to access dental care [12]may also contribute to poorer oral health. Appreciation of the importance of good oral health to overall well-being has increased as has evidence of inequalities in health for those in care homes [13]. Within this context, it is unsurprising that interest in how best to maintain and improve oral health among care home residents should increase.

It was within this context that the National Institute for Health and Care Excellence issued guideline NG48 [14] in 2016 that set out a series of recommendations for the improvement and maintenance of good oral health of care home residents. Subsequent reviews by the Care Quality Commission (CQC) [15] have highlighted improvements in awareness of the guidance by care home managers (from 61% of managers in 2019 to 91% in 2022), in adoption of policies to promote and protect residents’ oral health (from 25% of care home providers in 2019 to 53% in 2022) and in the use of oral health assessments upon admission (from 73% of care home providers in 2019 to 83% in 2022).The guidelines, however, are quite broad and not directly implementable, containing only a limited number of specific actions that could be utilised by care home staff. While the most recent CQC review noted more widespread practices regarding the review and update of resident care plans to reflect changing resident needs, it also noted a wide variation in the detail contained in such plans [15]. Perhaps this is unsurprising given the generality of guidelines and the wide variation in the organisational and structural characteristics of care homes across the country. As such, the evidence base in respect of interventions promoting oral health care in a care home environment remains sparse [16].

To advance understanding and pave the way for more definitive studies, a feasibility study entitled ‘Improving the Oral HealTh of Older People In Care homes (TOPIC)’ was undertaken to determine the potential of a definitive randomised controlled trial in this context [17]. The study examines the feasibility of a complex intervention framed around key aspects of the NG48. It consists of: (1) the administration by trained care home staff of the Oral Health Assessment Tool [18]; (2) a “support worker assisted” daily tooth-brushing regime with toothpaste containing 1,500ppm fluoride; and (3) a care home staff training package. The intervention was adapted using co-design principles and a suite of materials were produced based on the experience of those that provide care for older people in care homes [19]. The study is a pragmatic cluster randomised control feasibility trial conducted in 22 care homes across two locations– Northern Ireland and London. The results in terms of the feasibility and the process evaluation are presented separately [20, 21]. In parallel, a cost-consequence feasibility analysis explored the relevance and relative importance of different outcome measures in an assessment of value for money for an intervention of this type. Focus in this exercise was given to the feasibility of collecting what were considered to be key data and also on the strategies to efficiently collect such data for an evaluation. This paper reports the results of this exercise and discusses its implications for an economic evaluation to be run alongside a subsequent definitive trial.

## Methods

Quantitative and qualitative data were collected from residents and staff (managers) from the care homes that participated in the TOPIC study, and a range of key stakeholders that included care commissioners, registration and inspections bodies, resident advocacy (the voluntary sector) and community dental practitioners with experience of delivering care in care homes.

Quantitative data were collected directly from residents through researcher-administered questionnaires in care homes. These data were collected in 22 care homes across two sites (11 each in London and Northern Ireland). care homes were randomised to an intervention arm (*n* = 11) and a control arm (*n* = 11). Randomisation was in pairs on a 1:1 intervention/control ratio, using a dynamic adaptive randomisation algorithm with stratification by geographic location (Northern Ireland/ London). Ethical approval for this was received from the London: City & East Research Ethics Committee (ref: 19/LO/1107). All participants provided written informed consent. Residents were eligible to participate if they had capacity to provide consent and met the following inclusion criteria: aged 65 years and over; dentate or partially dentate; and living full-time in the care home. Residents were not eligible if they: were receiving end-of-life or palliative care; had severe cognitive impairment (6-item Cognitive Impairment Test (6-CIT) score of 10 or higher); or did not have a working level of spoken English.

The intervention comprised 3 elements. First, care home staff received a training package (written and online training) to raise awareness and provide skills to implement oral health promotion activities (the package was also used for the induction of new staff). Second, an oral health assessment tool was used to assess resident needs at baseline and 12 month follow-up and develop a personal oral healthcare plan for each resident. The oral health assessment tool [18] comprised a brief and practical assessment of the resident’s oral health needs that is reviewed and updated over time based on guidance within NG48. Third, a support worker assisted twice daily in resident tooth-brushing with 1500ppm fluoride toothpaste. Care homes allocated to the control arm continued usual care over the same 12 month period. Further details are available elsewhere [20] and in the trial protocol [17].

Data on health-related quality of life was collected from residents using the EQ5D5L [21] (at baseline, six months and twelve months follow-up), and on oral health-related quality of life through the Oral Impacts on Daily Performances (OIDP) [22] at the same time points. Data from care home staff were collected using a short survey circulated by email to participating homes organized in the October of 2023. The survey solicited information across a range of topics intended to inform the feasibility study. Areas covered were whether there were records of dental treatments provided to residents; whether such data were readily available and whether they contained details of treatments provided and by whom; arrangements for provision of oral healthcare training for staff; whether the care home maintained records of residents with particular oral health needs and; what care home managers perceived to be key costs and outcomes from their perspective.

These data were supplemented with data collected in interviews with key stakeholers conducted in December of 2023, as noted above, that covered perceptions of the challenges for conducting a cost-consequence study of an oral health promotion intervention in a care home environment and the value of such a study. Each interview began with a brief discussion of the importance of oral health in a care home environment and impressions of the existing evidence base on the effectiveness of interventions in this area. An open conversation then followed in which respondents were asked to reflect on what they saw as the key challenges around the delivery and evaluation of an intervention in this area. When the issue was not raised by the respondent, interview prompts referred to the implications for staff, given other commitments; the implications of staff turnover; the availability of data and challenges around collecting data; the outcomes they considered important and; the usefulness of an intervention and its evaluation for improving oral health in this environment. Respondents were free to discuss any other issue they thought important. Interviews were conducted by phone/online and typically lasted 45 min to one hour. The study was undertaken in a manner consistent with CONSORT guidelines for pilot or feasibility studies.

### Data analysis

EQ5D5L captures quality of life across 5 domains, with 5 levels of severity in each domain. For each timepoint quantitative data on EQ5D5L were tabulated as percentages across levels of each dimension of the framework. No attempt was made to apply preference weights to EQ5D5L scores as agreed preference weights are currently unavailable [21] and this is in any event beyond the scope of the feasibility study. Attrition– the change in sample size over time - and changes in responses were examined to assess the potential threat that attrition might pose to an economic evaluation in a subsequent definitive study. Data related to the OIDP are reported elsewhere [20]. Survey data collected in care homes were reported as percentages across response items before being synthesized narratively.

Five interviews were undertaken - due in part to the feasibility of interviewing experienced individuals across the UK in a relatively short time window (2 months). Contemporaneous notes were taken during the course of the interviews. These were used immediately after each interview to construct a thematic record of the conversation, recording the responses of interviewees to prompts (when these were used), to note issues they raised independently and the language they used to describe issues related for example, to time pressure facing staff in care homes. Findings were shared with interviewees who had the opportunity to amend the record.

## Results

### Resident survey of health-related quality of life

A total of 119 residents were recruited to the study, of which 115 provided usable outcomes data at baseline (four residents did not complete baseline after consent). Of those who provided usable EQ5D5L responses, 60.9% were female, the average age was 84 years and 30% (of those who provided responses) were exempt from dental charges. Details of the recruitment, retention and data completion rates for the study are provided elsewhere [20].

In Table 1 results from the EQ5D5L survey are presented by time point– baseline (t0), 6 months (t1) and 12 months (t2) follow-up - in terms of percentage by domain. Three points are worthy of note with respect to the results. First, attrition is evident over time with the number of completed responses falling from 115 to 78 between t0 and t2. The attrition rate differs between sites; in Northern Ireland 50% of responses were missing at t1 and a similar amount at t2. By contrast in London approximately only 20% were missing at t1 and 38% at t2. Attrition will affect all data collected, not just the EQ5D5L, though non-response may differ across individual items depending on how challenging respondents find completing a particular survey. Second, it is evident from the responses that health deteriorated over time. For example, with respect to those reporting issues with mobility the percentage who reported no problems in walking fell from 26.1 to 14.1% between t0 and t2. With respect to self-care the fall was even more evident, the percentage reporting no problems washing or dressing falling from 48.7 to 16.7% between t0 and t2. Similar patterns were evident across domains with a shift in the distribution toward greater problems in health. Third, the domain profiles indicated that residents were more likely to experience mobility issues, followed by self-care, issues with usual activities, pain/discomfort and anxiety/depression being less prevalent at moderate or above levels.

In Table 2, socio-demographic characteristics of respondents are reported. At baseline 63 of the sample were drawn from control homes and 52 from intervention homes. As can be seen most residents identified as being from the British Isles, as being widowed, and the overall average age was 84. More granular detail is not provided to avoid potential disclosure. No attempt was made to compare responses across groups as the study was not powered to detect differences.

### Survey of care home managers

With respect to the survey of care home staff, 7 care homes supplied data– no data was returned from care homes in Northern Ireland– and all homes were from the intervention group. All homes reported that they routinely record information about dental care provided to residents, and 4 were able to furnish examples of dental care in some detail. Five homes reported on what type of practice provided dental care; NHS and private providers were mentioned with equal frequency, with 2 homes reporting that dental care was provided by exclusively private providers, 2 exclusively by NHS and 1 by NHS, private and community dental services.

Two homes reported staff training in delivery of oral hygiene taking more than 20 min; 3 homes reported that it took 10–20 min and 2 reported that training took less than 10 min. Five homes reported that 25–50% of their residents had some of their own teeth; 1 home reported that it was more than 75% and 1 home less than 25%. All care homes reported that oral hygiene was provided to residents by care staff– though 1 qualified this by also saying “no one” suggesting that when under time pressure staff may have to prioritise tasks other than oral hygiene.

Four aspects of time, as a resource, were offered to managers as being potentially impacted by the intervention: time needed to train care assistants; time taken to supervise care assistants in delivery of an intervention; time taken by care assistants to deliver the intervention and; time saved for care assistants in not having to manage poor oral health. Managers were asked to rank order aspects of time as factors that would influence their decision to adopt an intervention. Looking at the average sum of ranks indicated that, from a management consideration, the impact on care assistant time for delivering the intervention and/or potentially time saved in managing the outcomes of poor oral health were ranked as equally important, followed by time needed to train staff.

Four aspects of outcomes were offered to managers as being potentially impacted by the intervention: residents’ quality of life related to everyday activities such as eating; residents’ quality of life related to pain; improvements in the resident’s oral health; and improvements in residents health generally. Managers were asked to rank order these as factors that would influence their decision to adopt the intervention. The average sum of ranks indicated that improvements in residents’ everyday quality of life were deemed most important followed by improvements in health generally. Five out of 7 homes ranked first the residents’ quality of life related to everyday activities such as eating. In terms of staff turnover, five homes estimated it to be between 0 and 10%, 1 at 11–20% and 1 at 21–30%.

### Stakeholder interviews

With respect to stakeholder interview, two interviewees were from a registration/inspection background (A & B); one was from a community dental service background (C ); one was from a service commissioning background (D) and one was from a patient advocacy background (E). Four of those interviewed (A-D) had trained as dentists though not all currently practiced dentistry. A number of themes recurred across the interviews with key stakeholders. First was the importance of oral health to overall health and well-being and the importance of prevention in particular given challenges around access to dental care. While all respondents mentioned the importance of prevention, informant C elaborated, noting the challenges for more advanced treatment in this patient group. The occasional need for general anaesthesia because of compliance issues (related to cognitive impairment), was specifically mentioned as giving rise to additional risks and costs that could be avoided through appropriate prevention. At the opposite end of clinical need, informant E, argued that the importance of intervention for social interaction should not be underestimated, independent of its clinical effects. It was suggested that what was referred to as a “placebo” in terms of oral health may nonetheless have important quality of life effects, and this should not be discounted.

Second, the challenging circumstances that care home staff work under and the implications of these both for the delivery and evaluation of an intervention was referenced by all informants. There was a general perception that staff-resident ratios were low, resident needs complex and priorities other than delivering oral hygiene would be more pressing. A perception of high staff turnover was also uniformly referenced and seen as adding to the demands on staff. As argued by informant B, undertaking steps to avoid bed ulcers may be prioritised over tooth brushing by staff. This may be especially the case if, as referenced by informants A and C, staff may be reluctant to brush teeth either because of the intimacy involved or issues of consent with residents.

Third, the pressure on dentists in providing care to residents and for residents in accessing care from dentists was widely referenced. Four informants (A-D) referenced this issue and the implications for oral health in care homes arising from it. Informant A highlighted regional inequalities in access to publicly funded care provided by general dental practitioners, with dependence on the private sector as a result. While informants A and B expressed some concerns with regard to the range of treatments that the general dental practitioner service could deliver due to issues of transporting equipment (oxygen for example), the main issue appeared to be the opportunity cost of providing such care given current levels of remuneration. Informants C and D, noted that community dental service staff were also under considerable pressure, with competing demands from other sources - those with learning disabilities being mentioned explicitly. Interestingly the competing needs of those with learning disabilities was echoed by informants E and A. As with comments related to the complexity, cost and risk of treatment in this age group, these underscored the value of prevention in this population.

Fourth, the importance of developing a set of evidence based standards against which performance in care homes can be assessed was referenced. Perhaps unsurprisingly the two informants (A and B) from registration and inspection backgrounds both made reference to the importance of a standard grounded in evidence to inform their inspections. These stakeholders, provided useful insights into the conduct of inspections and the constraints within which inspections were undertaken, in particular with respect to time pressure on inspectors. They commented that while time was taken to speak with staff, residents and family members, there was no attempt to undertake physical examinations of residents (“they do not look in people’s mouths”). It seemed unlikely that any future inspection would include physical examination of residents. In consequence inspections of adherence to new guidelines would likely rely on documentary evidence maintained by homes as well as interviews with staff, residents and family members. Informant D, a service commissioner, also referenced the need for an evidence base to help inform commissioning while informant C referred to a “manifest need” for research to provide clear direction on what works. These comments highlight an appreciation that however standards are operationalized, if they are to be inspected there needs to be an appreciation of the time constraints within which inspectors operate and what is feasible in consequence.

Other issues alluded to by informants included concerns regarding participant sample study attrition (informant C) and the potential care home (informant B) related for example to bankruptcy that could again affect the feasibility of an evaluation. These echo in some respects the point raised for the EQ5D5L data above.

## Discussion

The aim of this study was to assess the feasibility of conducting a cost-consequence evaluation of an oral hygiene intervention within a care home context. The sine qua non for such a study is the need to develop an evidence base for its effectiveness and cost-effectiveness. Based on the literature [16]the reviews of NG48 [23] and the comments received from informants interviewed as part of this study, it is clear that there is a manifest need for the development of an evidence base. The variability evident in current practice identified in the NICE review for NG48 [23], will as suggested by key stakeholders, delay the development of standards against which behaviour in homes can be assessed. This in turn will hinder the adoption of preventative measures at a time when it is clear oral health is an issue that impacts residents in a manner disproportionate to their community dwelling peers, challenging homes and the providers of dental treatment that are already under pressure.

This study demonstrates that it is possible to collect key data on outcomes from within the recruited care homes. That both homes and residents of homes were willing and able to engage with the research augurs well for the conduct of a definitive trial suggesting that it is feasible to collect the evidence necessary to assess the value for money of an oral health intervention though it is acknowledged this will not be without its challenges. While care home staff were aware of dental treatments provided to residents, not all homes were able to describe such data. This suggests that such data would need to be collected by researchers to ensure the veracity and accuracy of its measurement. Given the time constraints under which care home staff operate this would seem especially warranted but presents no obstacle in principle to the conduct of a definitive trial.

The study, however, does highlight issues that need to be carefully navigated in the conduct of an economic evaluation alongside a future definitive trial. The survey of care homes undertaken as part of this study took place in October 2023 towards the end of the study and when homes in Northern Ireland– which had undergone a change in ownership/management - may have been winding down their involvement with it. That less time was afforded for its completion and for follow-up with non-responders may help explain the poor response. In retrospect conducting the survey sooner would have been wiser and something that could be factored into a definitive trial if access to care home records or personnel is important. The data collected nevertheless suggests that care home managers are acutely aware of the potential for an oral hygiene intervention to reduce the burden on care assistants and improve the quality of life of residents– issues to which they appear to attach a high value to. In this regard it echoes the reviews of NG48 [23]. This is important as it suggests conditions are conducive for recruitment of homes to a future study. That most homes had a significant number of dentate residents, were aware of the dental care that residents received, the time required to train staff and had in place arrangements for providing assistance with tooth brushing again provides evidence that a definitive trial is feasible. However, as the completion rates for these data were less than optimal and the data provided by some homes was vague collecting such data prospectively rather than relying on retrospective records or memories of staff may be a more prudent approach.

The study also highlights challenges for the conduct of an evaluation alongside a definitive trial related to attrition. The high attrition rate among residents recorded here should not be surprising given the age and frailty of those at whom the intervention would be targeted. This could present challenges for data imputation if an intention to treat approach is adopted. Missing data, however, is unlikely to present an insurmountable issue. Published guidance on handling of missing data within evaluations alongside trials is available [24]. The EQ5D5L data produced in the study appears to have good face validity both in regard to the domains of health that exhibited limitations and how this changed over time. The percentage of subjects reporting no issues across domains of health declined though this should be interpreted carefully given the potential for survivor effects. That is, the exit of sicker members of the original cohort through death or inability to provide responses may mask to some extent the decline in health of survivors through changes in the composition of the cohort. Attrition notwithstanding, as might be expected in this population a decline in health over time is evident but the transition was to poorer health rather than the most severe health state, indicating that the decline was not precipitous, among survivors. In other words, data would exhibit change and be available for a significant number of participants over the course of a relatively short period. While the issue of attrition in this population presents issues for a definitive study, the existence of a proxy version of EQ5D5L that has been deployed in a care home environment [25] provides a contingency plan for a future study. With appropriate contingencies and transparent recording of the reasons for attrition, this should not present an insurmountable problem for a definitive study. As noted elsewhere, OIDP data were also available suggesting both cost-utility and cost-effectiveness calculations would be feasible.

An issue of perhaps greater concern is how generalisble the results of a study based on those able to give consent in a care home environment is to those unable to give consent. The Alzeimar’s Society estimate that in the UK about 70% of people in care homes have dementia or severe memory problems [28]. In most homes such persons likely make up a large group of residents who stand to benefit from an intervention of the type envisaged in NG48. While, as noted, proxy measures could be used for those unable to give consent or unable to respond for other reasons, studies that have examined the validity and feasibility of using proxy measures of health-related quality of life are rare. One study noted low levels of missing data, adequate levels of internal consistency and no evidence of floor effects in measures collected using EQ5D5L by proxy [29]. This could be taken as offering some comfort to the feasibility of a study based on a broader population of residents. Another study, however, that examined agreement between proxy and self-reported measures, again using EQ5D5L, was less positive [30]. This reported the strength of agreement to be fair between resident and proxy reported measures in the case of only one domain of EQ5D5L and in the index scored measure of quality of life but only slight agreement in respect of other domains or visual analogue data. This suggests that it may be more difficult to capture the evidence of effects for an intervention on this group using trial data. Recourse may have to be made to other proxy data, such as use of dental services, prescriptions for antibiotics or analgesia from which inferences might be drawn if access to it can be agreed. This is though something a definitive trial will need to consider.

Similarly, for those residents who can provide consent to access care home and dental records, there is no reason that the issue of missing data or attrition in respect of resource use could not be overcome, at least to an extent. It should be relatively easy, that is, for a research team to identify, record and measure resource use in the care home using an adaptation of the client services receipt inventory [26]. This data could be supplemented from records within the home and/or dentists’ records of those consented to a definitive trial and sources cross referenced to provide a more complete and in places verified record– provided access can be agreed, including those for for whom consent is an issue. While the existence of multiple sources of funding for personal and dental care will add to the complexity of monetizing resource use, this is a common issue in mixed payment systems and when a broader perspective than that of a publicly funded payer is adopted. That private general dental practitioners will likely charge higher fees than publicly funded dentists and may also be more easily accessed, is not in itself a significant barrier to collection of resource use data or its monetization. This may similarly be the case with respect to care assistants responsible for the delivery of an intervention. At a minimum, it suggests a sub-group analysis comparing private and public residents/dentist users may be warranted in a future evaluation. This, however, is a relatively minor issue which can be addressed with little effort. It does, however have implications for the perspective that a definitive study should adopt. While NICE recommends a NHS and personal social services perspective [27]– what might be interpreted as a publicly funded perspective– this could produce anomalies in this particular context as it may not be the reality for all residents. For example, the potential savings/costs associated with an intervention would be related to the percentage of residents whose care (including dental care) is funded publicly. This will vary between homes and is likely also to vary across the UK. To avoid this the adoption of a societal perspective may be more straightforward alongside a NHS perspective with the suggested sub-group analysis providing useful additional insights.

The lack of engagement with the survey of care home managers in Northern Ireland sounds a cautionary note about management of relationships over the course of a trial. Had the survey been conducted at the outset of the feasibility study it is probable that a larger number of responses would have been obtained as additional time would have been afforded for completion and additional time for follow-up with care homes who did not respond. By the same token, however, care home managers will have had more informed opinions at the end of the study than at the start which must also be considered. Clearly, a balance here has to be struck as to when information is sought but ongoing engagement with homes throughout a study will be important.

The study has a number of strengths and limitations. Surveying residents, care home managers and interviewing relevant stakeholders allowed responses to be triangulated from distinct perspectives as regards the feasibility of undertaking a cost-effectiveness and cost-utility evaluation in a definitive trial. Arguably this provided a more rounded assessment of the feasibility than would otherwise have been the case as well as identifying the challenges that might be encountered and how these might be overcome. Demonstrating the feasibility of collecting outcomes data related specifically to quality of life in a care home environment provides concrete evidence that a research team can recruit homes and collect data of the type needed for an evaluation of a definitive oral health trial. This augurs well for the collection of the type of quantitative data– on a range of outcomes and costs– that would be needed to populate a cost-consequence model. That care home managers from Northern Ireland did not engage with the survey of managers is a limitation though one that could be explained and overcome. While attrition was evident among residents, this is not unsurprising for a subject group of this type and does in itself jeopardize a definitive trial. The issue of generalizability to the broader care home community is something that a definitive study will need to carefully consider.

## Conclusions

Overall, the study supports the case for the conduct of an economic evaluation in a definitive trial to address a manifest gap in the evidence base on oral hygiene interventions in a care home context. It identified quality of life as a key outcome of concern to stakeholders including care home managers. It demonstrated the feasibility of collecting such data with face validity among those able to consent in this context. Thus a case is made for a definitive study to adopt a cost-utility approach of the type favoured by NICE for an evaluation. A parallel cost-effectiveness analysis based on OIDP would also be prudent, as would a close examination of the correlation between any outcomes and use of services. The study suggests that among those care homes who responded, data on provision of oral hygiene and utilization of dental treatments could be readily collected by a research team and validated by reference to dental records. It identified care assistant time as a key resource likely to influence the decisions of care home managers when assessing the success of an intervention. The existence of high attrition rates and the mixed economy of care homes– with private and publicly funded personal and dental care services– present challenges for the conduct of an economic evaluation alongside a definitive trial, as does the generalisabilty of the findings. The evidence collected in this study, however, suggest that with careful planning these should not be insurmountable issues.

**Table 1:** Results of EQ5D5L survey Percentage by response category

Time		Domain				
	Level	Mobility	Self Care	Usual activity	Pain/Discomfort	Anxiety/depression
Baseline	1	26.09	48.70	73.04	72.17	69.57
	2	18.26	16.52	9.57	12.17	14.78
	3	13.04	8.70	7.83	10.43	12.17
	4	21.74	9.57	5.22	5.22	3.48
	5	20.87	16.52	4.35	0	0
						*N* = 115
6 month	1	19.48	24.68	38.96	51.95	49.35
	2	9.09	10.39	18.18	16.88	18.18
	3	24.68	19.48	19.48	18.18	24.68
	4	20.78	23.38	19.48	11.69	7.79
	5	25.97	22.08	3.90	1.30	0
						*N* = 77
12 month	1	14.10	16.67	26.92	50.00	46.15
	2	20.51	20.51	28.21	16.67	32.05
	3	28.21	30.77	26.92	25.64	17.95
	4	17.95	14.10	10.26	6.41	2.56
	5	19.23	17.95	7.69	1.28	1.28
						*N* = 78

Note level refers to level of severity, where 1 is lowest– for example in mobility, I have no problems walking about– and 5 is highest, for example in mobility I am unable to walk about. Responses were drawn from intervention and control groups. No attempt was made to make comparison by group

**Table 2:** Socio-demographic characteristics of resident respondents to quality of life survey at baseline

	Control (*N*)	Intervention (*N*)	All (*N*)
Male	21	24	45
Female	42	28	70
			*N* = 115
Eng/Wel/Scot/NI/Britain/ Ireland	56	46	102
Other^*^	7	5	12
			*N* = 114
Never married	13	10	23
Widowed	37	25	62
Other^**^	12	17	29
			*N* = 114
Mean Age (standard error)	85.60 (1.01)	82.36(1.27)	84.13 (0.81)
			*N* = 115

## Data Availability

Data are available from the corresponding author upon reasonable request.

## Declarations

### Permission to reproduce material from other sources

N/a.

### Clinical trial registration

ISRCTN10276613. Registered on 17th April 2020. http://www.isrctn.com/ISRCTN10276613.
